# Case report: A rare case of thyrotropin-secreting pituitary macroadenoma with diffuse calcification presenting with hyperthyroidism and literature review

**DOI:** 10.3389/fonc.2023.1121140

**Published:** 2023-02-16

**Authors:** Huiying Yan, Chaolong Yan, Jiannan Mao, Wei Jin

**Affiliations:** Department of Neurosurgery, Nanjing Drum Tower Hospital, The Affiliated Hospital of Nanjing University Medical School, Nanjing, China

**Keywords:** pituitary adenoma (PA), TSHoma, hyperthyroidism, calcification, macroadenoma

## Abstract

**Background:**

Thyroid-stimulating hormone (TSH)-secreting pituitary adenomas (TSHomas) are rare and usually present with hyperthyroidism. Calcification in pituitary tumors is an infrequent finding. Herein, we report an extremely rare case of TSHoma with diffuse calcification.

**Case description:**

A 43-year-old man was admitted to our department with a complaint of palpitations. An endocrinological examination revealed elevated serum levels of TSH, free triiodothyronine (FT3), and free thyroxin, whereas the physical examination revealed no obvious abnormality. Computerized tomography (CT) showed a sellar mass with diffuse calcification. Contrast-enhanced T1-weighted images revealed a less-enhancing tumor without obvious suprasellar or parasellar expansion. The tumor was completely removed *via* endoscopic transnasal-sphenoidal surgery. Microscopically, nests of cells were inconspicuous among the diffuse psammoma bodies. Expression of TSH was patchy, and only several TSH-positive cells were observed. Postoperatively, the serum levels of TSH, FT3, and FT4 decreased to their normal range. Follow-up MR images showed no evidence of residual tumor or regrowth after the resection.

**Conclusions:**

Herein, we report a rare case of TSHoma with diffuse calcification that presented with hyperthyroidism. A correct and early diagnosis was made according to the European Thyroid Association guidelines. This tumor was completely removed *via* endoscopic transnasal-transsphenoidal surgery (eTSS), and thyroid function was normalized after the operation.

## Introduction

Among all functional pituitary adenomas, thyroid-stimulating hormone (TSH)-secreting pituitary adenomas (TSHomas) are rare and account for only 0.5%–3% of all pituitary tumors in surgical series (1, 2). TSHomas can produce excessive amounts of TSH, resulting in increased levels of thyroxine, and present with clinical features of hyperthyroidism (3). Calcification in the pituitary tumor is an infrequent finding and has been radiologically found in two forms: eggshell-like capsular calcification and intratumoral nodular calcification. Until now, only about 50 cases of calcification in pituitary adenomas have been reported, and diffuse calcification is rare (4, 5). Herein, we report an extremely rare case of TSHoma with diffuse calcification that presented with hyperthyroidism in our department.

## Case presentation

**Figure 1:**
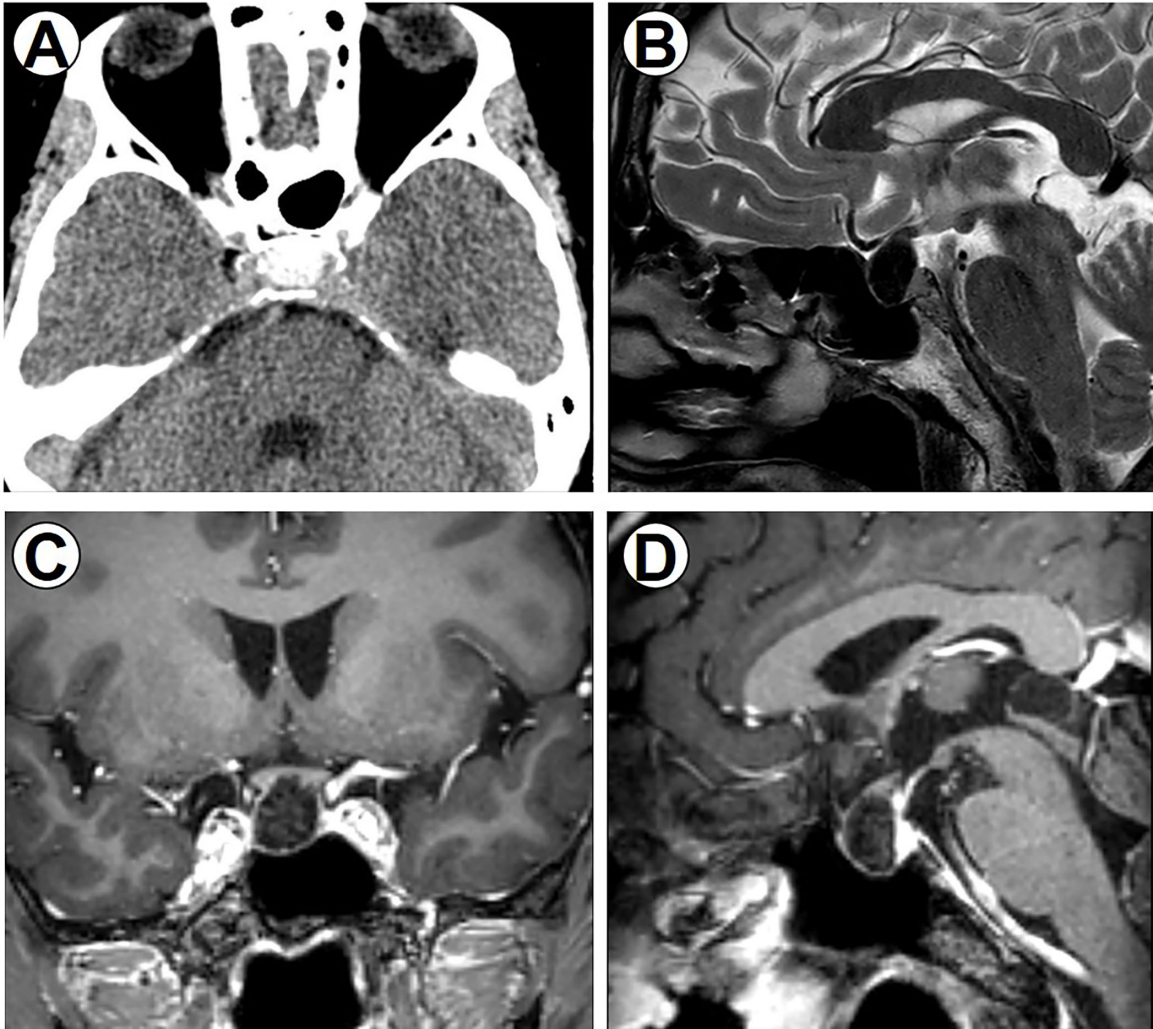
Perioperative imaging data. **(A)** Preoperative CT scan of the brain, axial section showing a sellar tumor with obvious calcification. **(B)** T2-weighted sagittal image showing hypointense sellar lesion. **(C)** Coronal T1-weighted image. **(D)** Sagittal T1-weighted image with gadolinium contrast showing hypointense sellar lesion anterior to the pituitary gland without enhancement.

A 43-year-old man visited the outpatient department complaining of palpitations. His height was 174 cm, and his body weight was 66 kg. He had continuously perspired profusely. His blood pressure was 160/95 mmHg. No abnormal findings were noted on physical examination, including the thyroid gland. Electrocardiogram showed atrial fibrillation, and the heart rate was 97 beats/min. Endocrinological examination revealed hypersecretion of TSH, free triiodothyronine (FT3), and free thyroxine (FT4); as a result, central hyperthyroidism was in consideration (**Table 1**). The thyroid iodine uptake was within the normal range. Other pituitary and adrenal hormones were within normal ranges. A sellar mass with calcification was then observed during computerized tomography (CT) scanning (**Figure 1A**). Magnetic resonance imaging (MRI) showed a sellar lesion without obvious suprasellar or parasellar extension. On enhanced scans, the tumor showed no enhancement after gadolinium administration (**Figures 1B–D**). The tumor size was 1.1 cm × 1.3 cm × 1.5 cm. The pituitary gland was compressed backward. The cavernous sinus and optic chiasma were not invaded. Short-term somatostatin analog test was performed (6), and the serum TSH concentration decreased by 53% after octreotide administration (**Table 2**). The examinations of autoantibodies and antineutrophilic cytoplasmic antibodies (ANCA) were all negative. Ultrasound detection of the thyroid revealed diffuse disorders; the thyroid was swelling without any mass detected, suggesting thyroid hypersecretion. To distinguish the syndrome of pituitary resistance to thyroid hormone (PRTH) (7, 8), the thyroid hormone receptor genes were examined, and there was no mutation.

**Table 1:** Serum levels of TSH and thyroid hormones.

	TSH (0.27–4.2 mIU/L)	FT3 (3.1–6.8 pmol/L)	FT4 (12–22 pmol/L)
**On admission**	5.19	15.11	52.21
**3 days before surgery**	3.37	11.9	42.3
**1 day after surgery**	0.437	6.74	39.3
**5 days after surgery**	0.083	3.23	24.8
**10 days after surgery**	0.144	2.36	16.5
**1 month after surgery**	0.829	3.21	9.25

**Table 2:** Somatostatin sensitivity test.

	TSH (0.27–4.2 mIU/L)
**0 h**	3.33
**2 h**	2.48
**4 h**	1.79
**6 h**	1.69
**8 h**	1.56
**24 h**	1.57

**Figure 2:**
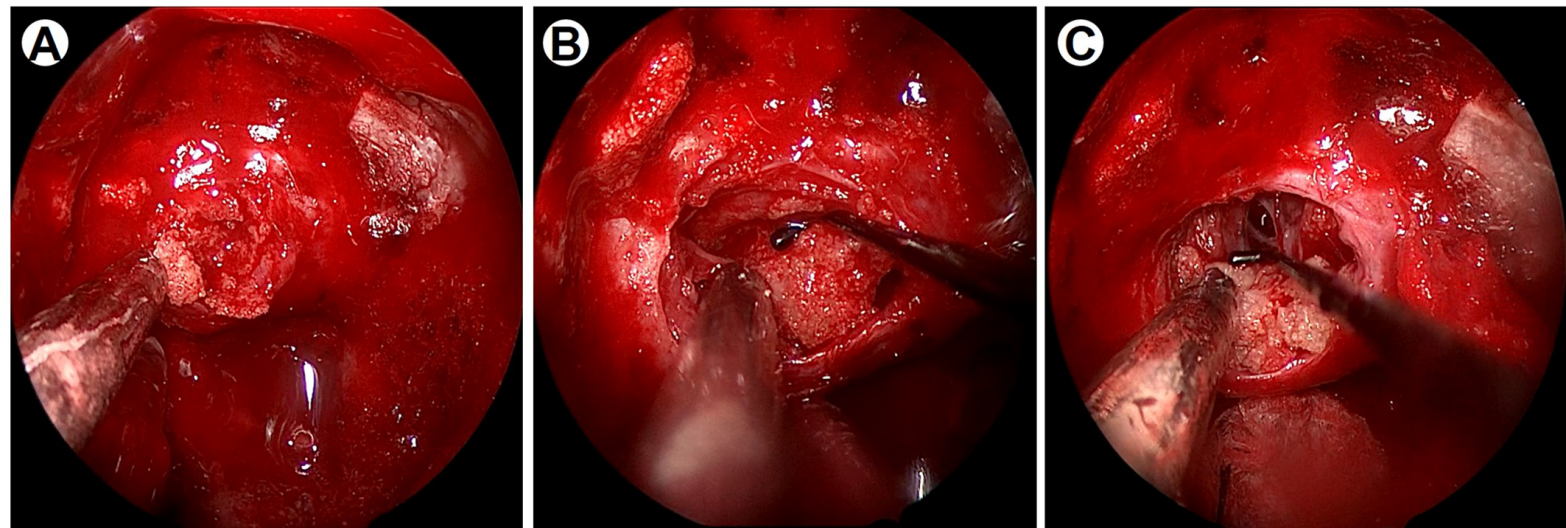
Intraoperative imaging data under neuroendoscopy. **(A)** Intraoperative imaging indicates the yellowish tumor was firm and consisted of psammoma bodies. **(B)** The lesion adhered closely to the diaphragma sellae. **(C)** After the tumor removal, the diaphragma sellae was broken and cerebrospinal fluid leakage was observed.

To prevent a thyrotoxic crisis during the perioperative period, thiamazole was administered at a dosage of 30 mg/day, and the hyperthyroidism was significantly relieved. After careful preoperative evaluation, endoscopic transnasal-sphenoidal surgery (eTSS) was performed in December 2020. During the operation, a solid yellowish mass full of calcifications was identified in the sellar area (**Figures 2A–C**). The mass was limited, and it did not appear to invade the cavernous sinus or diaphragma sellae. Total removal of the mass was achieved after eTSS.

**Figure 3:**
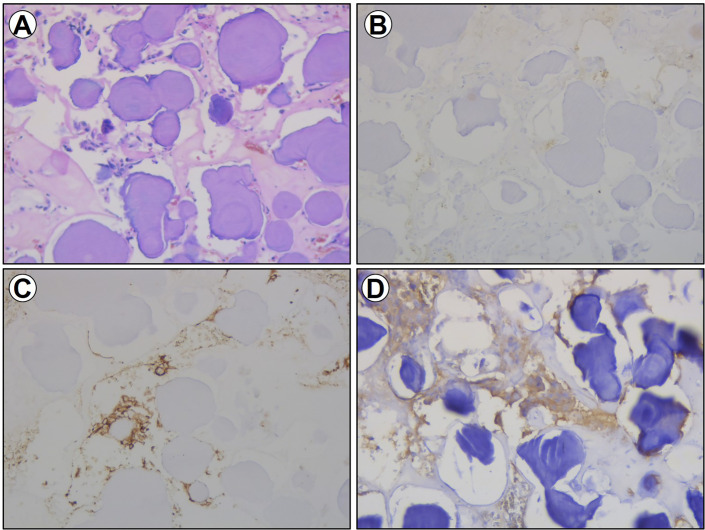
Section imaging data of the excised specimen. **(A)** HE staining for the excised specimen. **(B)** Immunohistochemical staining of TSH for the excised specimen. **(C)** Immunohistochemical staining of CD56 for the excised specimen. **(D)** Immunohistochemical staining of Syn for the excised specimen.

Histologic sections of the surgical specimen revealed an abundance of psammoma bodies, and nests of cells were inconspicuous among the psammoma bodies (**Figure 3A**). There was no evidence of pleomorphism, mitosis, or necrosis, suggesting a benign tumor. Upon immunohistochemical staining, only a few TSH-positive cells were detected. Moreover, the cells were negative for all other anterior pituitary hormones except follicle-stimulating hormone (FSH), epithelial membrane antigen (EMA), progesterone receptor (PR), P63, P40, and Ki67, but the cells were positive for chromogranin A (CgA), CD56, cytokeratin (CK), and synaptophysin (Syn) (**Figures 3B–D**).

**Figure 4:**
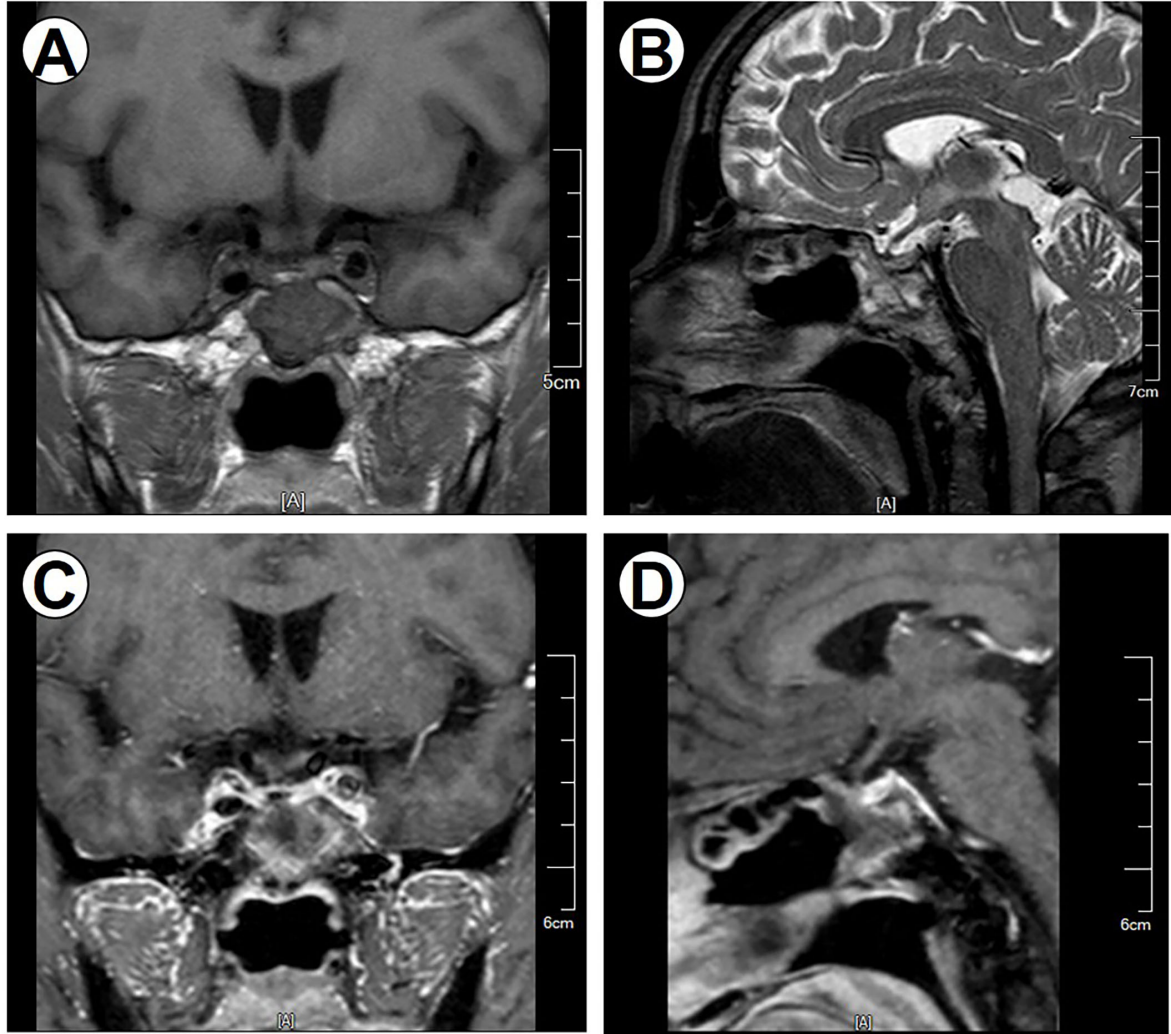
Postoperative MRI imaging data. Postoperative MR images demonstrate no regrowth of the tumor. **(A)** Coronal noncontrast T1-weighted image. **(B)** Sagittal T2-weighted image. **(C)** Coronal contrast T1-weighted image. **(D)** Sagittal contrast T1-weighted image.

Postoperatively, normalization of thyroid hormones was achieved with no deficiency of other pituitary hormones. The patient was discharged from the hospital on the 12th postoperative day. A follow-up MRI was performed 12 months after eTSS **(** **Figure 4** **)**; no regrowth of the tumor was noted, and the patient’s thyroid function tests were within normal limits.

## Discussion

TSHomas are the least frequent pituitary adenomas; they occur in all age groups and exhibit no gender predilection (2). TSHomas can be defined by serum hormone levels suggestive of central hyperthyroidism before surgery or by immunohistochemical profiles for TSH on postoperative pathological analysis. Some tumors that stain immunochemically for TSH are clinically silent or are associated with hypopituitarism (9, 10). The diagnosis of TSHomas is challenging. A correct and early diagnosis of TSHomas is essential to avoid misdiagnosis, and thereby, proper treatment could be taken immediately (11). Clinical manifestations of hypopituitarism, including sinus tachycardia, hypertension, and weight loss, were observed in this case. His endocrinological examination revealed hypersecretion of TSH, FT3, and FT4, suggesting the possibility of central hyperthyroidism (12). According to the European Thyroid Association guidelines (6, 13), a pituitary MRI/CT scan, short-term somatostatin analog test, and examination of thyroid hormone receptor genes were performed, and the results supported the diagnosis of TSHoma.

Surgical resection is considered the first-line therapy for TSHomas (1, 11), aiming at removing tumor mass and normalizing thyroid function. According to a recent meta-analysis, biochemical remission is achieved in about 70% of the TSHomas; however, total resection is slightly lower, at only 54% (14). This might due to the fact that most TSHomas are invasive macroadenomas (15). After careful evaluation and preoperative preparation, the eTSS was performed. In this case, TSHoma was noninvasive. During the surgery, we found that this tumor was hard and fixated. Moreover, the tumor adhered closely to the diaphragma sellae, and after the tumor removal, cerebrospinal fluid leakage was observed. Without considering endocrine abnormalities, this mass was more like a diaphragma sellae meningioma, especially a psammomatous meningioma (16). Fortunately, total resection was achieved, and thyroid function was normalized in this patient.

In accordance with practice, a histopathological examination was performed after surgery. Frequently, TSHomas are chromophobic and the tumors are usually composed of irregular or elongated angular cells possessing long cytoplasmic processes, and TSH immunoreactivity is variable (10, 17). Among pituitary stones, most of them are presented in the form of psammoma bodies scattered between adenoma cells. In the current case, diffuse psammoma bodies were observed at light microscopy, and nests of cells were inconspicuous around them. After decalcification, immunohistochemistry of the resected specimen showed expression of TSH was patchy and only several TSH-positive cells were observed. Moreover, TSH is also frequently expressed in plurihormonal secretory adenomas. In our case, the cells were positive for FSH, while other circulating hormones were in the normal range.

The sellar region, one of the most complex neoplastic areas of the brain, can give rise to a wide variety of pathologically distinct tumors, including pituitary adenoma, germ cell tumor, Rathke’s cleft cyst, meningioma, chordoma, craniopharyngioma, etc. (18). Most of these tumors, especially craniopharyngiomas, are often accompanied by calcification; however, pituitary adenomas with calcification are scarcely rare (19). According to previous reports, calcification in pituitary adenomas could be radiologically observed in two forms: eggshell-like capsular calcification and intratumoral nodular calcification. The latter, also known as “pituitary stones,” are relatively rare. Furthermore, calcification in pituitary adenoma raises special diagnostic and therapeutic challenges (20). In this case, the tumor was diffusely calcified on a CT scan, and it was hard to get the correct diagnosis of pituitary adenoma just according to imaging studies. Generally, gross total resection *via* eTSS approach is considered difficult to accomplish in pituitary adenomas with extensive calcification because of the hardness. With the development of neuroendoscopic surgery, some calcified pituitary adenomas could be completely removed *via* the eTSS approach, even when large, as in the current case. The eTSS approach might be an effective and feasible option for removing such tumors after careful evaluation.

The real mechanisms of calcification formation in pituitary adenomas were still unclear. Previous studies suggested that pituitary apoplexy, hyperprolactinemia, regressive processes of pituitary adenoma, and treatment by dopamine receptor agonists or irradiation might be the possible causes (4). These hypotheses were based on cases in which the persistence of prolactin granules was detected in the calcified adenomatous tissue (21). However, it also appears that calcification can occur in any type of pituitary adenoma, including prolactinoma, suggesting that hormone secretion might not be the main mechanism of pituitary stone formation. Apart from endocrine abnormalities, calcification was also found in some cases with intratumoral hemorrhages or degenerative changes during surgery or pathological examination (21). Nevertheless, without these findings or in the absence of the medical histories described above, calcified pituitary adenomas have also been reported. In this case, no evidence suggesting the mechanism of calcification formation was found. This result suggested that other causes might be involved in the calcification formation in pituitary adenomas.

## Conclusions

TSHoma is a rare type of functional pituitary adenoma and often presents with hyperthyroidism. A correct and early diagnosis of TSHomas is challenging; it can tell the surgeons to provide proper treatment. Calcification in pituitary tumors is an infrequent finding and identification of calcification before the operation is essential as it helps in surgical planning. Herein, we report a rare case of TSHoma with diffuse calcification that presented with hyperthyroidism and was completely removed *via* an eTSS.

## Data availability statement

The raw data supporting the conclusions of this article will be made available by the authors, without undue reservation.

## Ethics statement

Written informed consent was obtained from the individual(s) for the publication of any potentially identifiable images or data included in this article.

## Author contributions

HY and CY wrote and revised the manuscript together. JM participated in the surgery and handled the pictures. WJ gave some important advice for the revision of the manuscript. All authors contributed to the article and approved the submitted version.
